# Integration of CRISPR/Cas9 with artificial intelligence for improved cancer therapeutics

**DOI:** 10.1186/s12967-022-03765-1

**Published:** 2022-11-18

**Authors:** Ajaz A. Bhat, Sabah Nisar, Soumi Mukherjee, Nirmalya Saha, Nageswari Yarravarapu, Saife N. Lone, Tariq Masoodi, Ravi Chauhan, Selma Maacha, Puneet Bagga, Punita Dhawan, Ammira Al-Shabeeb Akil, Wael El-Rifai, Shahab Uddin, Ravinder Reddy, Mayank Singh, Muzafar A. Macha, Mohammad Haris

**Affiliations:** 1 Department of Human Genetics-Precision Medicine in Diabetes, Obesity and Cancer Program, Sidra Medicine, Doha, Qatar; 2 Saha Institute of Nuclear Physics Complex (MSA-II), Kolkata, West Bengal India; 3 Department of Pathology, Michigan Medicine, University of Michigan, Ann Arbor, MI USA; 4 Vector Laboratories, Inc, 6737 Mowry Ave, Newark, CA 94560 USA; 5 Department of Biotechnology, School of Life Sciences, Central University of Kashmir, Ganderbal, Jammu & Kashmir India; 6 Laboratory of Cancer Immunology and Genetics, Sidra Medicine, Doha, Qatar; 7 Department of Medical Oncology, All India Institute of Medical Sciences, New Delhi, India; 8 Department of Surgery, Miller School of Medicine, University of Miami, Rosenstiel Med Science Bldg., 1600 NW 10Th Ave, Room 4007, Miami, FL 33136-1015 USA; 9 Department of Diagnostic Imaging, St. Jude Children’s Research Hospital, Memphis, TN USA; 10 Department of Biochemistry and Molecular Biology, University of Nebraska Medical Center, Omaha, NE USA; 11 Translational Research Institute, Hamad Medical Corporation, Doha, Qatar; 12 Laboratory Animal Research Center, Qatar University, Doha, Qatar; 13 Center for Advanced Metabolic Imaging in Precision Medicine, Department of Radiology, Perelman School of Medicine, University of Pennsylvania, Philadelphia, USA; 14 Watson-Crick Centre for Molecular Medicine, Islamic University of Science and Technology, Awantipora, Jammu & Kashmir India

**Keywords:** CRISPR/Cas9, Artificial intelligence, Genome engineering, Cancer precision medicine, Cancer Immunotherapy, CAR T-cells, Epigenetics, Drug resistance, Cancer biomarker

## Abstract

Gene editing has great potential in treating diseases caused by well-characterized molecular alterations. The introduction of clustered regularly interspaced short palindromic repeats (CRISPR)/CRISPR-associated protein 9 (Cas9)–based gene-editing tools has substantially improved the precision and efficiency of gene editing. The CRISPR/Cas9 system offers several advantages over the existing gene-editing approaches, such as its ability to target practically any genomic sequence, enabling the rapid development and deployment of novel CRISPR-mediated knock-out/knock-in methods. CRISPR/Cas9 has been widely used to develop cancer models, validate essential genes as druggable targets, study drug-resistance mechanisms, explore gene non-coding areas, and develop biomarkers. CRISPR gene editing can create more-effective chimeric antigen receptor (CAR)-T cells that are durable, cost-effective, and more readily available. However, further research is needed to define the CRISPR/Cas9 system’s pros and cons, establish best practices, and determine social and ethical implications. This review summarizes recent CRISPR/Cas9 developments, particularly in cancer research and immunotherapy, and the potential of CRISPR/Cas9-based screening in developing cancer precision medicine and engineering models for targeted cancer therapy, highlighting the existing challenges and future directions. Lastly, we highlight the role of artificial intelligence in refining the CRISPR system's on-target and off-target effects, a critical factor for the broader application in cancer therapeutics.

## Introduction

**Fig. 1:**
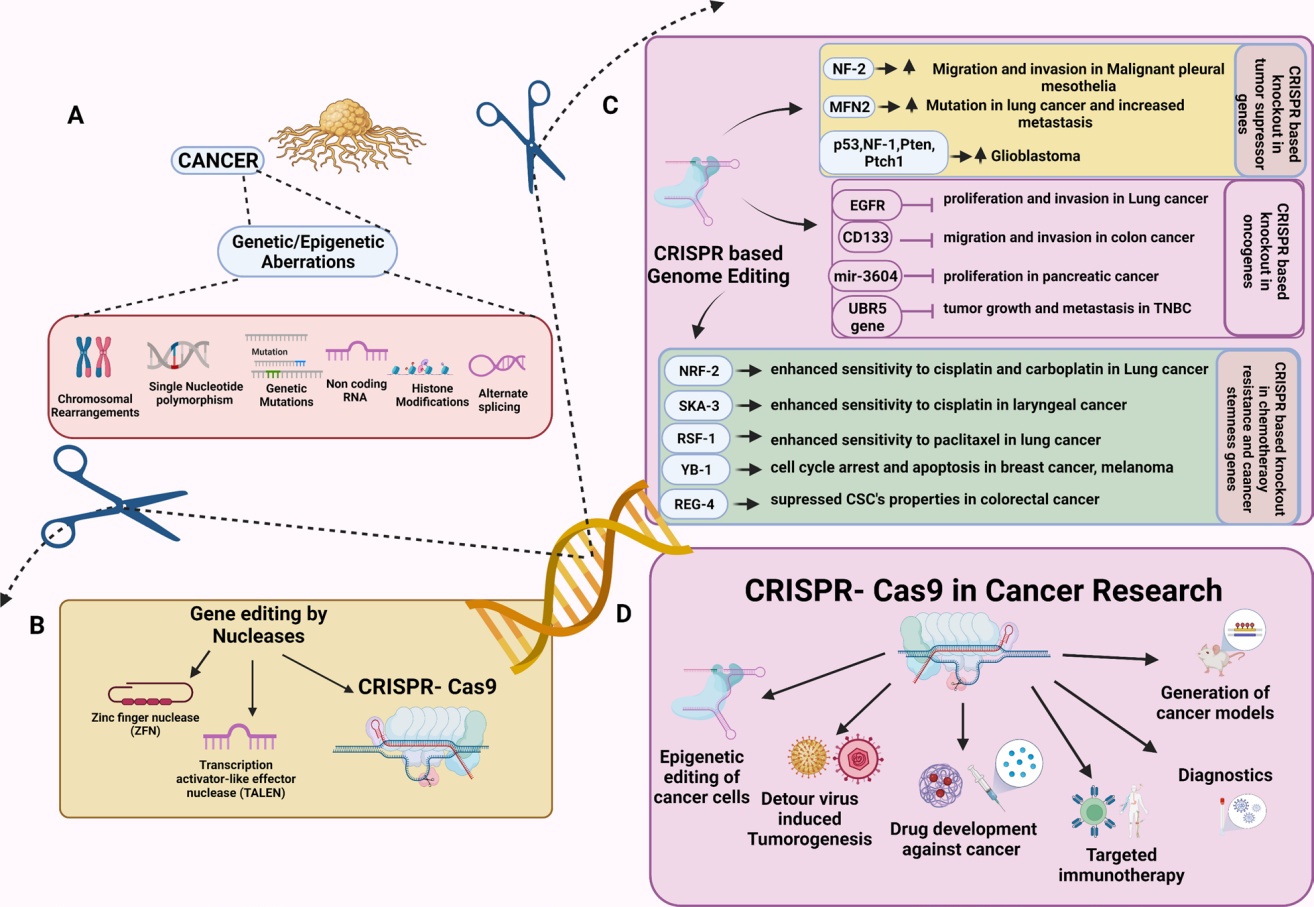
CRISPR/Cas9 in cancer research. **A** Schematic diagram illustrating cancer initiation and progression by involvement of multiple genetic and epigenetic alterations in cancer. **B** Different approaches used for genome editing in cancer include ZFNs, TALENs, and CRISPR/Cas9 systems. **C** CRISPR/Cas9 editing targets specific genes or growth factors regulating oncogenic processes. **D** Numerous mutations and dysregulated expression of oncogenes, tumor suppressor genes, chemotherapy-resistant genes, and cancer stem cell–related genes involved in tumorigenesis targeted by CRISPR/Cas9 system can be used for discovery of novel biomarkers and therapeutic targets in cancer research

As our understanding of the underlying genetic and molecular basis of malignancy has rapidly increased through massive tumor genetic profiling, modeling, and characterization, the ever-evolving list of molecular alterations in cells holds great potential for identifying actionable genomic events and treating malignancies. The emergence of gene-editing tools in the last few decades has enabled scientists to manipulate genomic sequences to understand gene function better and develop targeted treatments for inherited and acquired diseases. Although the 1970 discovery of restriction enzymes, the original genome editor, was a breakthrough enabling the recognition of specific nucleotide sequences, the use of restriction enzymes was limited due to their inability to direct targeted DNA cleavage at specific sites [1]. Efforts to improve the accuracy and specificity of restriction endonucleases led to the discovery of mega nucleases such as zinc-finger nucleases (ZFNs) and transcription activator-like effector nucleases (TALENs). These engineered nucleases have facilitated genetic manipulation by inducing targeted double-stranded breaks (DSBs), culminating in the activation of either of the two major cellular DNA repair mechanisms, the non-homologous end joining (NHEJ) or homology-directed repair (HDR) (Fig. 1) [2].

The discovery of the clustered regularly interspaced short palindromic repeats (CRISPR)/CRISPR-associated protein 9 (Cas9) system as a genome-editing toolbox significantly transformed the editing of genomic targets by enabling researchers to manipulate genomic elements efficiently and precisely. Initially reported in the prokaryotic genome as part of an antiviral defense mechanism against bacteriophages, the CRISPR/Cas9 system was later recognized as a revolutionary genome-editing tool enabling insertion, removal, and deletion of existing genes with high precision and specificity [3].

**Fig. 2:**
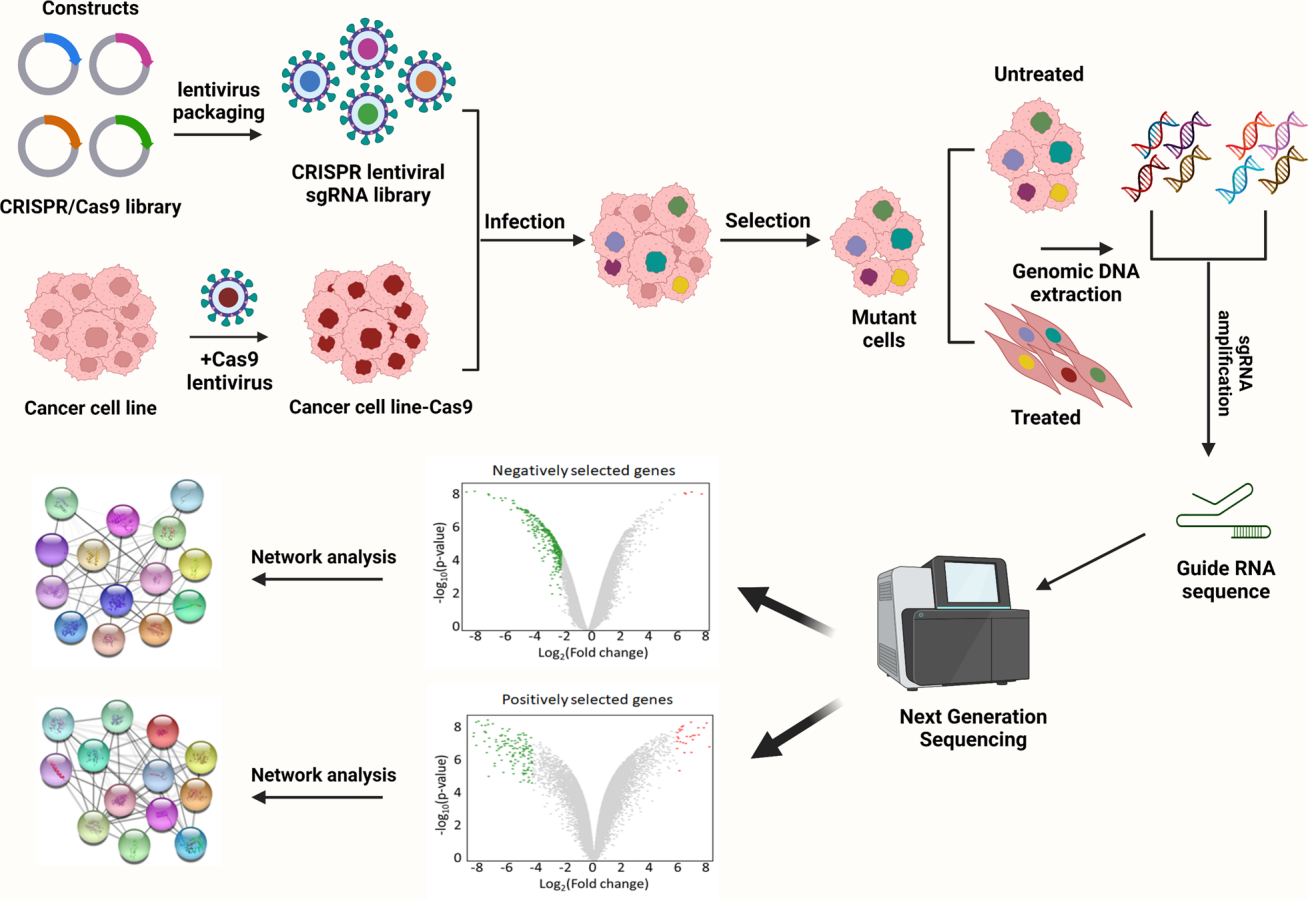
Schematic workflow of genome-wide CRISPR/Cas9 screening. A human genome-wide CRISPR/Cas9 knock-out library with sgRNAs is packed into lentiviral particles and transduced into Cas9-overexpressing cancer cells. The sgRNA-transduced cells are selected to generate mutant cells. Mutant cells are treated with drugs and DMSO (vehicle). DNA is extracted, and sgRNA is amplified via PCR. Whole-genome screening is conducted via next-generation sequencing before bioinformatics analysis. Volcano plots depicting genes selected with and without drug treatment and the corresponding networks are shown, with enriched genes on nodes and signaling pathways highlighted

In the last few decades, several genes associated with cancer initiation and progression have been identified using high-throughput screening technologies such as next-generation sequencing (NGS) and whole-genome, -exome, and -transcriptome sequencing [4–6]. CRISPR/Cas9 has been a tool of choice for studying the function and regulation of specific genes and in high-throughput screening approaches (Fig. 2). However, the data generated from these high-throughput technologies require testing and validation using suitable genetic models to infer drug targets and develop efficient treatments. In this context, CRISPR/Cas9 has been the tool of choice for studying the function and regulation of those genes in valuable genetic models, such as isogenic cells, with the same genetic background. In association with sequencing technology, CRISPR/Cas9 has shown promise not only for testing and validating drug targets but also for identifying functional genes such as tumor suppressors, oncogenes, drug resistance genes, cancer stem cells (CSCs), and cancer metabolism–related genes, thus improving our understanding of cancer initiation and progression, a critical step in developing precision treatments (Fig. 1) [7]. To facilitate the success of clinical therapeutics in the drug discovery process, validation of the drug target is a necessary and crucial step. Early drug target validation enables an increased understanding of the effect of target manipulation on disease biomarkers and disease endpoints and the clinical spectrum of the disease. The identification of functional mutations that confer drug resistance is considered the gold standard for drug target engagement and confirmation. In this regard, the high specificity and the ability of the CRISPR/Cas9 system to efficiently manipulate gene targets allows the selection of ideal therapeutic drug targets, thus simplifying the process of drug target selectivity and validation [8–10].

As the next challenge, the potential of the CRISPR/Cas9 system to correct these cancer-associated aberrations should be a concerted effort in cancer therapy with improved on-target and reduced off-target effects. This review discusses the applicability of the CRISPR/Cas9 system for genome engineering in cancer research and treatment, along with addressing the various algorithms that have greatly improved the efficacy of the system in clinical therapeutics.

## The evolution of CRISPR/Cas9

In 1987, Ishino et al. reported homologous sequences of 29 nucleotides arranged as direct repeats with 32 nucleotide spacers in the alkaline phosphatase isozyme conversion gene of *Escherichia coli* [11]. In 2002, with the advances in DNA sequencing technology, similar repetitive DNA sequences were found in archaea and bacteria by using in silico analysis [12]. These sequences are termed 'clustered regularly interspaced short palindromic repeats (CRISPR) because of their characteristic structural features that include the presence of direct repeats (21–50 bp) interspaced by non-repetitive sequences or spacers beside CRISPR-associated (Cas) genes. CRISPR's unique spacer sequences are homologous with viral or bacteriophage sequences that infect bacteria and archaea, suggesting that they might be part of an adaptive immune system providing immunity against foreign nucleic acids [13–16].

In a bacterial CRISPR system, the Cas9 nuclease moderates the invading bacteriophage DNA cleavage, which is incorporated between CRISPR repeats as “spacers” that later act as genomic signatures of the pathogen. Upon subsequent bacteriophage invasion, spacers produce CRISPR RNA (crRNA) containing protospacer regions complementary to the foreign DNA. The crRNA hybridizes with a transactivating crRNA (tracrRNA) [17], encoded by the CRISPR system. The resulting hybrid crRNA-tracrRNA is then associated with the Cas9 nuclease, establishing a CRISPR/Cas9 system. The protospacer of the crRNA recognizes its complementary region on the foreign DNA, which is followed by its cleavage (adjacent to motif sequence “NGG”) by Cas9’s nuclease domain [18]. The double-stranded breaks (DSBs) generated by the RNA-guided Cas9 activate the DNA repair machinery via error-prone NHEJ, leading to random insertion, deletion, or mutation in the genome or via template-dependent HDR [19, 20]. In 2011, Sapranauskas et al. described the successful transfer of the CRISPR/Cas9 system from *Streptococcus thermophilus* to *Escherichia coli* [21]. These findings were essential to understanding the mechanism of the naturally occurring CRISPR immunity system and laid the foundation for establishing the CRISPR/Cas9 system as a genome-editing tool.

In 2012, the functional application of the CRISPR/Cas9 system was first carried out in vitro*,* demonstrating the role of crRNA in target sequence recognition and Cas9 protein-mediated DNA cleavage [19, 20]. In their study, Jinek et al. used a single guide RNA (sgRNA) designed by fusing the crRNA and tracrRNA sequences, an established feature of the CRISPR/Cas9 system. In 2013, three pioneering studies engineered a type II bacterial CRISPR/cas9 system to successfully edit genes using custom-designed RNA-guided nuclease activity in mammalian cells [22–24]. These studies marked the beginning of a paradigm shift in basic and clinical research as the CRISPR/Cas9 system provided researchers with a potent tool for targeting any desired genomic loci. In 2013, Qi et al. developed the catalytically dead dCas9 protein, deficient in endonuclease activity but able to initiate CRISPR interference (CRISPRi) and repress target genes [25]. Systems developed by Meader et al. have also fused the dCas9 with the VP64 transcriptional activation domain capable of initiating CRISPR activation (CRISPRa) to increase the expression of target genes [26].

Furthermore, multiple naturally occurring Cas proteins and their engineered variants, such as *Streptococcus pyogenes* Cas9 (SpCas9), SpCas9-VRER variant, SpCas9-NG variant, SpCas9 variant SpRY, SaCas9, CjCas9, xCas9 3.7, Cas12a, Cas13a, Cas13d, and dCas13, have been developed with different novel applications and target sequence recognition specificities, expanding the potential applications of the CRISPR/Cas9 technology (Table 1).

**Table 1:** CRISPR/Cas9 variants and their applications in cancer treatment

Tool	Cas9 fusion/	Cancer biology	Application	Ref.
CRISPRa	Cas9-SAM	Prostate cancer	Identification of genes associated with drug resistance	[53]
	Cas9-VP64	Immunotherapy	Precision targeting of mutated genes	[135]
	Cas9-VPR	Colorectal cancer	Models altered glycosylation associated with cancer	[136]
	Cas9-BCL-xL	Hematologic malignancies	Regulation of CAR-T cells	[137]
CRISPRi	Cas9-KRAB / Cas9-suntag	Myelodysplastic syndrome	Mechanism of action (Rigosertib)	[138]
	Cas9	Multiple myeloma	Mechanism of action (immunotherapy)	[139]
	Cas9-KRAB	Glioblastoma	Identification of lncRNAs as therapeutic target	[140]
	Cas9-KRAB	Squamous cell carcinoma	Suppression of oncogene ΔNp63	[141]
NGS	Perturb-CITE-seq	Melanoma	Define mechanisms of resistance	[39]
	scRNA-seq	Cutaneous squamous cell carcinoma	Roles for specific tumor subpopulation-enriched gene networks in tumorigenesis	[142]
	Spear-ATAC-seq	Leukemia	Identification of regulatory networks	[143]
	scRNA-seq (scRibo-STAMP profiling)	Triple-negative breast cancer	Identification of RNA binding protein as therapeutic target	[144]
Base Editor	Campylobacter jejuni CRISPR-associated protein 9-fused adenine base editor (CjABE)	Glioblastoma	Precision targeting of mutated genes	[145]
	BE3, BE4, and ABE	Leukemia	T-cell–based immunotherapy	[146, 147]
Knock in	Cas9	Esophageal squamous cell carcinoma	Development of cellular immunotherapies	[148]
	Cas9	Colorectal cancer	Patient-derived organoids	[149]
	Cas9	Glioblastoma	Understanding quiescent glioblastoma	[150]
	Cas9	Colorectal cancer	Identification of stem cell markers	[151]
Knock out	Cas9	Breast cancer	Generation of organoid cancer models	[66]
	Cas9	Colorectal cancer	Validation of cancer driver genes	[152]
	Cas9	Ovarian high-grade serous carcinoma	Generation of cancer models	[153]
	Cas9	Lung cancer	Generation of cancer models	[154]
	Cas9	Head and neck squamous cell carcinoma	Validation of the function of *NRF21* gene	[155]

## CRISPR/Cas9 for in vitro screening

Cancer research needs rapid and practical tumor models that can recapitulate multiple molecular events that drive tumor progression in a cell. In this context, the CRISPR/Cas9 system has been shown to assist in developing accessible and feasible in vitro models of mammalian cells that can help identify genes and signaling pathways underlying cancer development and recurrence. In vitro CRISPR/Cas9 screening involves loss-of-function (CRISPR), CRISPR/Cas interference (CRISPRi), and CRISPR/Cas activation (CRISPRa) screens. For example, loss-of-function screens (CRISPR) targeting nearly 18000 genes in melanoma cell lines have been described as assisting in identifying genes resulting in resistance to RAF inhibitors [27]. Similar studies have identified ENL (eleven nineteen leukemia) as a critical domain for leukemic transformation [28]. CRISPRi and CRISPRa screens initially demonstrated by Gilbert et al*.*, however, involve catalytically dead dCas9 to alter gene expression [29]. The CRISPRi uses dCas9 fused with KRAB repressor to repress targeted gene expression transcriptionally; yet, in CRISPRa, dCAS9 is linked to an activator to overexpress target genes. Further fine-tuning of these methods has led to the development of inducible systems that use doxycycline for dCas9-KRAB expression [30]. Variants such as the Cas12a system have facilitated the use of multiple genome editing technologies [31]. Such combinatorial gene knock-out screens can identify more than one target gene, thus providing valuable information on biological pathways, sensitivity, or resistance to drugs in cancer research.

## CRISPR/Cas9 for in vivo screening

In addition to in vitro screening, the CRISPR/Cas9 system is applicable for in vivo screening as well, involving both an indirect and autochthonous screening approach. In an indirect screen, immortalized cancer cell lines (constitutively expressing the Cas9 nuclease) are transduced with guide RNA libraries and then transplanted into animals for induction of tumor growth and metastasis [32]. Different delivery methods are used for direct in vivo mutagenesis, including lentiviruses and adeno-associated viruses (AAV9). A study by Chen et al. utilized the mouse genome-scale CRISPR/Cas9 knock-out library (mGeCKOa) containing 67,405 single-guide RNAs (sgRNAs) to mutagenize a non-metastatic murine non-small cell lung cancer (NSCLC) cell line and the resulting mutant cell pool was found to generate metastasis when transplanted into the flanks of immunocompromised mice [32]. It was observed that specific loss-of-function mutation accelerates tumor growth and metastasis, and CRISPR/Cas9 serves as an efficient tool for the assessment of phenotypic loss-of-function mutations in vivo [32]*.* On the other hand, in an autochthonous screen, guide RNA libraries and Cas9 are directly delivered to animal models using adeno-associated viruses (AAVs) to generate tissue-specific cancer models, such as those in the liver, lung, and brain [33, 34]. A study utilized CRISPR/Cas9 genome editing to map functional cancer genome atlas of tumor suppressor gene (TSG) variants mutated in human cancers in an autochthonous mouse cancer model [34]. In the study, Cre-inducible CRISPR/ Cas9 mice livers were pool-mutagenized using AAVs carrying a sgRNA library targeting mutated tumor suppressor genes (mTSGs) [34]. It was shown that immunocompetent mice that received the AAV-mTSG library developed complex autochthonous liver tumors and died within 4 months [34]. To reveal the mutational landscape of the tumors, molecular inversion probe sequencing was performed to generate a direct readout of the Cas9 generated variants [34]. Thus, the study demonstrated the significance of AAV-mediated autochthonous CRISPR screens for mapping a provisional functional cancer genome atlas of tumor suppressors, oncogenes, or any other genetic events associated with tumor evolution in vivo.

## CRISPR/Cas9-based screens

Our understanding of the molecular regulatory network in cancer has been further advanced by combining next-generation sequencing platforms with CRISPR/Cas9-based screens. In a standard CRISPR/Cas9 screen, the guide RNA, transcribed by the RNA polymerase III at a U6 promoter, lacks a poly-A tail, so it cannot be funneled into RNAseq analysis, limiting the understanding of molecular mechanisms as a result of genetic perturbation. Novel techniques such as Perturb-seq [35], CRISP-seq [36], and CROP-seq [37] were developed by merging single-cell RNAseq analysis with the CRISPR/Cas9 screen. In CRISPR-seq, a polyadenylated unique guide index and a fluorescent selection marker are included with the gRNA vector module [36]; in CROP-seq, a second gRNA module with a poly-A signal is used for RNAseq [37], and in Perturb-seq, a guide barcode with a poly-A tail is included in the construct.

Furthermore, cancer cells evading immune treatment poses a major therapeutic challenge. Recently, studies by Frankiegh et al. have combined Perturb-seq and epitope-seq (CITE-seq) [38] to investigate the genes involved in immune checkpoint resistance (Table 1). Patient-derived melanoma cells were targeted with 248 intrinsic immune checkpoint inhibitors, and resistance signature genes, single-cell transcriptomics, and 20 cell surface proteins were profiled in more than 200,000 cells. Among several targets, CD58 was identified as an essential factor conferring immune evasion ability [39]. The development of these CRISPR-assisted technologies to study complex cellular circuitry has provided an efficient framework for an in-depth investigation of oncogenic drivers in cancer cells.

## CRISPR/Cas9 in cancer research

**Fig. 3:**
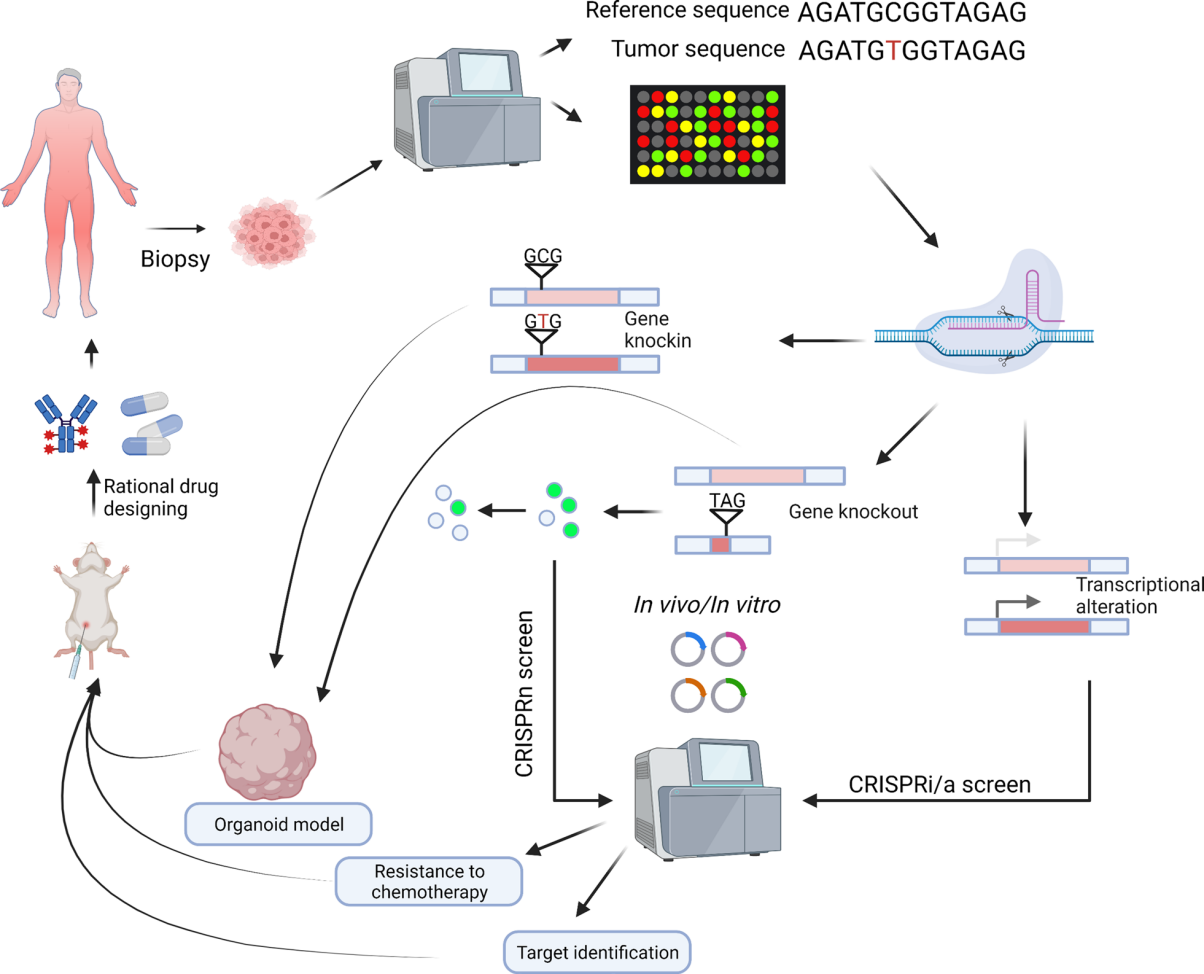
Implications of CRISPR/Cas9 genome engineering for personalized medicine in cancer treatment. Schematic showing the development of the CRISPR-Cas genome engineering platform to identify potential therapeutic targets and design cancer models specific to patient-specific genomic anomalies. CRISPR/Cas9-mediated knock-out, knockin or CRISPR Interference (CRISPRi) screens can be used to identify and validate novel drug targets, tumor-suppressor genes, cancer stem cell-related genes and to elucidate unknown drug resistance mechanisms, thus helping in perosnlized drug designing

Cancer is a highly complex disease where the "one size fits all" module of molecular characterization is insufficient for successful therapeutic intervention, necessitating personalized approaches to treatment for better prognostic outcomes for patients with cancer. The CRISPR/Cas9 system can be tailored to investigate gene functions via genome-wide screens and perform rational drug designing for targeted cancer therapy (Fig. 3). This section emphasizes the role of CRISPR gene-editing technology in characterizing cancer heterogeneity to enable precision medicine. CRISPR technology has been used to identify and validate novel drug targets and biomarkers, understand drug resistance mechanisms, and construct cancer models, which are critical areas for developing targeted therapeutic agents needed for precision cancer treatment.

### CRISPR-Cas9 base editing in cancer

Base editing provides an excellent platform for targeted gene editing by introducing specific base-pair alterations at programmable genome loci. Cytosine and adenosine base editors have been characterized and demonstrated to mutate C to T and A to G, respectively, in an sgRNA targeting–dependent manner [40]. Exploration of the clinical significance of these nucleotide variants in a high-throughput manner could facilitate our understanding of oncogenic transformation and expedite drug discoveries. Existing studies using base editing have laid the groundwork to define the functions of oncogenic mutations across different cancer models [40–43]. Because of the versatility of site-directed genome editing, we envision that base editing will be a potent tool for studying cancer genetic variants and will facilitate the development of precision-based medicine.

### CRISPR/Cas9-mediated drug targets

An essential goal of precision medicine in oncology is to identify critical drug targets dependent on specific genetic variants in cancer patients. The emergence of CRISPR/Cas9-based assays has revealed the power of gene manipulation to identify valuable proteins for therapeutic targeting.

Mixed lineage leukemia-rearranged (MLL-r) is associated with high-risk patient subgroups and shows an inferior prognosis [44]. CRISPR/Cas9-based negative selection assays revealed that MLL-r leukemic cell lines, such as MV411 and MOLM13, are susceptible to knock-out of a putative transcriptional regulator ZFP64, but non-MLL-r leukemic cell lines, such as K562 are not [45]. In another leukemia study, KAT7, an acetyltransferase that deposits acetyl moiety on lysine 14 and lysine 23 of histone H3, was identified as a critical target in leukemic cells harboring MLL-fusion proteins but not in leukemic cells with wild-type MLL. With the help of CRISPR/Cas9-mediated KO, the study correlated the loss of KAT7 to myeloid differentiation. KAT7 KO increased apoptosis in MLL-r leukemia cells, providing a breakthrough in novel target discovery based on the genetic makeup of hematological malignancies [46].

In addition to hematological malignancies, CRISPR/Cas9 has facilitated understanding solid tumor vulnerabilities dependent on genomic anomalies. In a cancer study of triple-negative breast cancer (TNBC), SUM159PT cells were used to represent the most aggressive genetic features of TNBC. CRISPR/Cas9-based screening for positive and negative regulators in the TNBC model study revealed an activated mTOR pathway and a suppressed Hippo/YAP pathway with the TNBC development [47]. ATRX, a member of the SWI/SNF chromatin remodeling complex, is frequently mutated in gliomas and liver cancer. Liang et al*.* performed a synthetic lethal CRISPR/Cas9 screen for ATRX deficiency in hepatocellular carcinoma (HCC) cell lines and identified WEE1 as a critical target [48]. The study has highlighted the significance of WEE1 targeting in cancer patients with ATRX deficiency. Furthermore, microsatellite instability (MSI) contributes to tumorigenesis in various tissues [49]. Hypermutable microsatellites arise due to the impairment of DNA mismatch repair systems and increase the susceptibility to developing cancer. Chan et al*.* have analyzed large-scale loss-of-function CRISPR screens to counter MSI susceptibility in cancer. These studies have reported that the RecQ DNA helicase WRN is more critical in MSI cancers than stable microsatellites, thus identifying an essential target in MSI-specific cancers [50].

### CRISPR/Cas9 role in drug resistance

Within a single tumor, subpopulations of cells have varied gene expression profiles that contribute to tumor heterogeneity, drug resistance, and subsequent tumor relapse. Understanding the resistance mechanisms is a primary challenge in targeted cancer therapeutics. The analysis of genetic alterations affecting the tumor-intrinsic activity of therapeutic agents in early development stages can transform the design of future clinical trials in terms of patient stratification strategies.

High-throughput loss-of-function CRISPR screens have enabled the identification of genes that confer resistance and synergistic lethal combination targets to address drug resistance. Shu et al. describe a CRISPR screen in the TNBC cell line to understand the mechanism of drug resistance treated with JQ1, a BET bromodomain inhibitor (BBDI), [51]. Follow-up studies have also revealed CDK4/6 kinase/microtubule inhibitors, such as paclitaxel combined with BBDIs, as potential candidates for treating resistant TNBC. These studies also show tumors with *BRCA1* and *BRCA2* mutations to be more sensitive to BBDIs [51]. Furthermore, Mo et al*.* have performed a CRISPR screen to understand the resistance mechanism to CC-122, a cereblon E3 ligase-modulating agent, in treating relapsed or refractory diffuse large B-cell lymphoma [52]. The study reported alterations in genes corresponding to NF-κB activation that can be used as predictive biomarkers of patients responding to CC-122 treatment [52].

Using an unbiased CRISPRa screen, Chen et al*.* reported that activation of cell-cycle checkpoint protein RAD9A increases regulatory T cells in the prostate tumor immune microenvironment, resulting in metformin resistance [53]. In a more comprehensive format, Schleicher et al*.* have established CRISPR and CRISPRa genome-wide screens to explore genes responsible for resistance to emerging anticancer agents such as Rad3-related (ATR) protein kinase inhibitors [54]. Specifically, the overlapping hits from the CRISPR KO screen on HeLa cells treated with ATR kinase inhibitors VE822 and AZD6738 were identified and validated. Later, CRISPR KO screens using VE822 and AZD6738 were performed in MCF10A and 8988 T cells. The genes identified from MCF10A and 8988 T cells were similar to those identified from the HeLa cells, confirming the genes identified from HeLa screens as being proper regulators of resistance to ATR inhibitors. Finally, CRISPRa screens using both VE822 and AZD6738 were performed in HeLa and MCF10A cells. Notably, the top hits overlapped between VE822 and AZD6738 treatment in each cell line, but the hits from HeLa cells did not match those of MCF10A cells [54]. This study’s results again highlight the role of CRISPR screens in identifying biomarkers and genetic signatures in determining the early stages of the development of drug resistance in patients with tumors.

### CRISPR/Cas9–based cancer models

CRISPR/Cas9 technology provides efficient genome editing and a more manageable approach to generating gain- or loss-of-function animal models, reducing the cost of developing genetically engineered mouse models for cancer studies [55]. The efficiency and simplicity of the technique have translated to several species, including *C. elegans*, zebrafish, pigs, and cynomolgus monkeys [56–59]. Here, we have focused on cell lines, organoids, and mouse models.

Chromosomal translocations caused by aberrant fusion of chromosomes often translate into an expression of novel fusion proteins and contribute to malignancies. Current models of tumor biology with chromosomal translocations rely on ectopic expression of fusion proto-oncogenes in in vitro cell lines or transgenesis and, thus, do not fully recapitulate the human disease states [60]. The emergence of CRISPR/Cas9–based genome editing has greatly facilitated the ‘engineering’ of such chromosomal events and testing their functional consequences in vitro and in vivo systems. One of the first clinically relevant human cancer model cell lines engineered with CRISPR/Cas9 involved a fusion between an echinoderm microtubule-associated protein-like 4 (EML4) and anaplastic lymphoma kinase (ALK) [termed as the EML4/ALK oncogenic fusion] that was engineered in non-small cell lung cancer (NSCLC) adenocarcinoma [61]. This fusion is associated with a response to EGFR kinase inhibitors. The ATCC has now developed an EML4-ALK fusion NSCLC cell line (ATCC® CCL-185IG™; www.atcc.org) that can be used to validate the detection of this rearrangement in patients with cancer to aid in forming a precise treatment plan. The engineered gene fusion mimics the spontaneous EML4/ALK rearrangements isolated from patients' tumors. It serves as a valuable model to screen novel ALK inhibitors and study tyrosine kinase signaling pathways mimicking this cohort. CRISPR/Cas9 can induce chromosomal translocation in human CD34^+^ hematopoietic stem cells to generate MLL-r–related hematopoietic malignant models [62, 63]. Similar models recapitulate relevant mutations in Isocitrate dehydrogenase 1 and 2 (IDH1 and IDH2) mainly found in low-grade gliomas, secondary glioblastomas, and acute myeloid leukemia (AML) [60], without the limitations of the earlier models. Similarly, constructs have been engineered with an IDH2 R140Q mutant allele to mimic AML progression due to genetic alteration and metabolic changes ([61]; www.atcc.org for IDH2R140Q).

Resistance to targeted therapy imposes a critical challenge in any clinical outcome, with patients’ tumors initially responding well but acquiring resistance through treatment. One prominent example is the resistance of melanoma cells to BRAF inhibitor therapy [62, 63]. The metastatic melanomas with BRAF V600E mutation are highly responsive to BRAF inhibitors (dabrafenib and vemurafenib) initially but later display resistance a few months post-treatment [64, 65]. This prompted developing a series of cell lines using CRISPR/Cas9 to introduce various clinically relevant point mutations associated with acquired BRAF inhibitor resistance into a BRAF V600E melanoma cell line ([65]; www.atcc.org). The BRAF V600E melanoma cell line was used as the parental cell line, and several clinically relevant point mutations associated with acquired BRAF inhibitor resistance were introduced into genes that act either upstream or downstream of BRAF in the Ras/Raf/MEK/ERK kinase signaling pathway ([112]; www.atcc.org).

In addition to conventional cell lines and animals, organoids can be genetically altered to model cancer. These self-organized, three-dimensional (3D) structures containing organ-specific cell types are grown from stem cells or organ progenitors in vitro. They are a valuable model system recapitulating the 3D structure, differentiated cell composition, and organ-specific function of primary human tissue [64]. CRISPR/Cas9 gene-editing technology is applied to organoids to generate complex cancer model systems to recapitulate tumor heterogeneity. Recently, Zhang et al*.* generated multiple high-grade serous tubo-ovarian cancer (HGSC) models by engineering mouse fallopian tube epithelial organoids via CRISPR/Cas9 genome editing [65]. Complex genetic models representing mutational combinations seen in patients, such as *Trp53*^*−/−*^*; Brca1*^*−/−*^*;Myc*^*OE*^ and *Trp53*^*−/−*^*;Pten*^*−/−*^*;* and *Nf1*^*−/−*^, have assisted in generating HGSC-like tumors. This study’s results suggest that MYC is an essential biomarker indicating PARP-I/platinum resistance in patients with HGSC. Moreover, these studies’ results suggest that patients with NF1-deficient tumors might benefit from a combination of paclitaxel with platinum [65]. Similarly, Dekkers et al*.* used CRISPR/Cas9 to knock out tumor suppressor genes, such as *P53, Pten, Rb1,* and *Nf1*, in normal human breast organoids generated from human reduction mammoplasties to mimic neoplasia [66]. The triple-mutant (*P53/Pten/Rb1)* and quadruple-mutant (*P53/Pten/Rb1/Nf1)* organoids successfully generated tumors upon xenotransplantation. Interestingly, these tumors responded to endocrine and chemotherapy, suggesting the potential utility of these organoid models in understanding and developing targeted therapies for breast cancer subtypes [66].

Murakami et al*.* have applied a genome-wide CRISPR KO screening approach in gastric organoids generated from mice to elucidate the mechanistic regulators of WNT-driven stem cell-dependent epithelial renewal [67]. This study identified multiple genes, including *Alk*, *Bclaf3*, and *Prkra,* that may suppress stemness/proliferation and function as novel regulators of gastric epithelial differentiation [67]. More importantly, these studies have opened up newer avenues for using CRISPR screens to generate custom-made tumor models in patient-derived organoids, thus assisting in devising effective ‘tailor-made’ therapies for patients with cancer.

### CRISPR/Cas9 in cancer immunotherapy

**Fig. 4:**
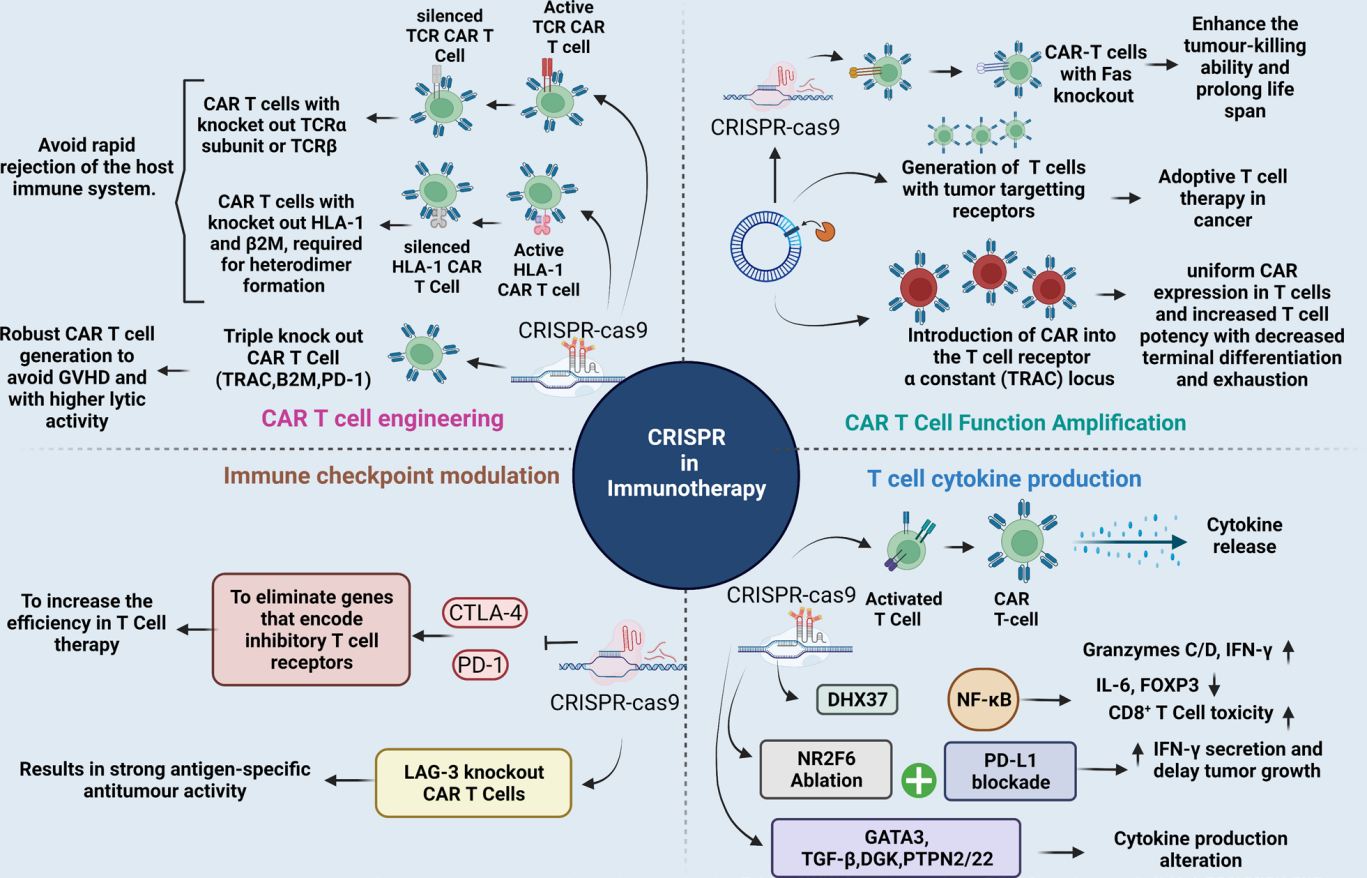
CRISPR/Cas9 in immunotherapy. The application of CRISPR/Cas9 system in editing CAR-T cells: **A** CRISPR/Cas9 system can be used to engineer CAR T-cells to make them more specific and to generate allogeneic universal CAR-T cells with reduced graft-versus-host disease (GVHD) responses. CRISPR/Cas9 system can simultaneously and efficiently knock out multiple gene loci to yield allogeneic universal T cells by incorporation of multiple guide RNAs in a CAR lentiviral vector, **B** CRISPR/Cas9 system can improve CAR T-cell functionality by avoiding off-target effects and making them more robust for enhanced proliferation and efficiency **C** CRISPR/Cas9 system can knock out inhibitory molecules (immune checkpoints) to enhance function of CAR-T cells, and **D** CRISPR/Cas9 system can modulate T-cell cytokine production to reduce the risk of cytokine release syndrome and inflammation for enhanced efficiency of cancer therapeutics

Immunotherapy assists the immune system in fighting infections and diseases. It mainly involves monoclonal antibodies, immune checkpoint inhibitors, T-cell transfer therapy, vaccines, and other immunomodulatory therapeutic drugs (Fig. 4) [68]. However, these drugs/treatments are not equally effective in all cohorts of patients. Moreover, their effectiveness has been found in combinatorial treatment with chemotherapy and other traditional approaches, such as radiation therapy and surgery [68]. However, the hallmark of recognition came with the 2018 Nobel Prize in Physiology for immune checkpoint inhibitors and their application as bi- and tri-specific mAbs [69].

Moreover, with the CRISPR/Cas9 system, a whole new Pandora’s box opened up, giving physicians and patients more combinatorial approaches to gene therapy to explore ( Fig. 4) [70]. The first CRISPR/Cas9 human trial in 2016 resulted in wider choices for patients, with multi-faceted, long-lasting benefits, as the production of therapeutic immune cells increased in patients. One example is the construction of CAR-T cells and programmed cell death protein 1 (PD-1) knock-outs. Thus, CRISPR/Cas9 technology may offer a much-desired treatment option in cancer immunotherapy.

### CRISPR/Cas9 clinical trials

The importance and demand for CRISPR were recognized in 2020, with its creators’ receiving the Nobel Prize. The current CRISPR-based treatment trials are still in their early stages, focused on the safety and side effects of the method. Ongoing trials cover five treatment portfolios (35 clinical trials recruiting, withdrawn or suspended, and completed using search words CRISPR/Cas9): blood disorders, cancers, eye disease, chronic infections, and protein-folding disorders (ClinicalTrials.gov). Clinical trials such as *NCT04601051* in patients with Hereditary Transthyretin Amyloidosis and Polyneuropathy and *NCT03872479* address rare genetic disorders causing childhood blindness [71] and have been widely discussed.

In the oncology portfolio, search words cancer and CRISPR/cas9 reveal 20 clinical trials, of which two were completed in China, 4 have been withdrawn or suspended, and 14 are recruiting. The first-in-human phase I clinical trial (NCT02793856) used PD-1 knock-out engineered T cells generated using the CRISPR/Cas9 tool for treating patients with metastatic NSCLC. This study recruited NSCLC patients in 2016 [72] who received PD-1–edited T cells for the next five years [73–75]. Notably, the study did not include CAR-T cells but used a CRISPR/Cas9 mediated knock-out of the *PD-1* gene (an immune checkpoint inhibitor) in T-cells. The salient features of this trial are as follows: safe, tolerable, desired edit found in a median of 6% of T cells/patient before infusing back into patients; low-frequency of off-target effects; on-target effects with a median of 1.69%. The most-characteristic feature was that 11 of 12 patients had edited PD-1 T cells at low levels, even after two months of infusion. The success rate varied based on patients’ levels of edited T cells: the higher the level, the less the disease progression. The CARs are engineered with synthetic receptors that redirect lymphocytes (T cells) to a specific target antigen [76]. The success of anti-CD19 CAR-T cell therapy against B-cell malignancies and its approval by the U.S. Food and Drug Administration in 2017 [76] opened new avenues of treatment in combination with CRISPR/Cas9 and immune checkpoint inhibitors [76, 77].

Similarly, the first CRISPR-based therapy trial in the United States (NCT03399448) combined CAR-T and PD-1 immunotherapy approaches, using CRISPR to make a triple edit of three genes: two encoding the T-cell receptors [TCRα (TRAC) and TCRβ (TRBC)] and the third encoding the immune checkpoint inhibitor (PD-1) [77, 78]. The deletion of TCRα and TCRβ helped to reduce the mispairing of TCR and enhance the expression of a synthetic, cancer-specific TCR transgene, giving it CAR-T characteristics. Overall, the adoptive transfer of these engineered T cells (at all three genomic loci) into patients was found to have long-lasting effects and displayed therapeutic success. This was part of a Phase 1 study that began recruiting in 2018 and was completed in February 2020. As with the Chinese trial, the aim was to determine the safety, tolerance, and side effects, if any. Of the three patients who volunteered for treatment, two had advanced white blood cell cancer (myeloma), and one had metastatic bone cancer (sarcoma). The salient features of this trial were as follows: treatment was safe and tolerable; T cells went to bone marrow and remained there for nine months; TILs were found in tumor; low-frequency of off-target effects, and 70% of cells displayed at least one mutation at/near target site. Interestingly, the percentage of cells with mutations decreased over time, indicating that mutant cells were dying or out-competed by other cells.

Increasing numbers of pre-clinical and clinical studies involve the CAR-natural killer (NK) cells [79, 80], showing better prospects than CAR-T cells because of their more-robust antitumor response with fewer side effects. In addition, the choice comes from the unique mechanism of NK cells to distinguish pathologic cells from normal tissue cells as they are innate immune effector cells and lack antigen-specific receptors. In addition, they abundantly express neural cell adhesion molecule (NCAM; also known as CD56) and recognize a wide range of ligands on target cells [79]. Significantly, CRISPR-Cas9 gene editing in CAR-NK cells can help to counteract tumor-initiated immunosuppressive effects. Currently, efforts are in place to develop a good manufacturing practice–compliant strategy to produce off-the-shelf, CRISPR-modified, cord blood-derived CAR-NK cells to treat patients with cancer [81].

The success of the CRISPR/Cas-9 and CAR-T clinical trials and CAR-NK pre-clinical and clinical trials opens a gateway for developing novel frontline treatments. Limitations include toxicities, inhibition, and resistance in B-cell malignancies; limited efficacy against solid tumors; exhaustion; antigen escape; tumor infiltration; homing of TILs; and the immunosuppressive tumor microenvironment. Nevertheless, they are the subject of ongoing studies [76]. CRISPR-based therapies currently aim to treat blood cancers such as leukemia and lymphoma, and NSCLC [82, 83] shows higher response rates and persistent response, especially with studies involving biomarkers [84]. However, editing genes directly in the body would open new avenues for treating a greater range of diseases, making "treating the untreatable” possible [85].

## Future of CRISPR/Cas9

As the field of precision medicine is rapidly evolving, advancements are being made to develop personalized treatments based on a patient’s risk of disease or predicted response. Novel and emerging personalized medicine approaches, such as genome editing technologies using CRISPR/Cas9, can allow genetic modifications tailored to treat various Mendelian and complex diseases. Most CRISPR/Cas9 studies are performed in pre-clinical settings (in vitro and ex vivo studies). The system’s implementation in clinical settings is challenging owing to editing efficiency and off-target effects [86]. The off-target effects occur when Cas9 binds to and cleaves unintended genomic binding sites, resulting in abnormal gene function [87]. However, some of the latest approaches/strategies, such as cytosine or adenine base editors, ribonucleoprotein delivery, truncated g-RNAs, prime editing, and selection of different Cas variants, can minimize the off-target effects of the CRISPR system [87].

Recently, human induced pluripotent stem cells (hiPSCs) have been extensively used to represent human disease models as they exhibit phenotypes that closely mimic human pathologies [88]. The CRISPR/Cas9 system and hiPSCs could be helpful in drug development and screening, gene therapy/gene editing, and as a therapeutic immune response strategy against viral infections [89, 90]. Patient-specific induced pluripotent stem cells, with high replicative capacity and pathogenic genetic alterations, can be utilized to study the underlying molecular mechanisms in complex genetic disorders. However, this strategy is the genetic manipulation of the pluripotent human cells that prevents the generation of efficient genetically defined disease models [91]. Furthermore, in contrast to ZFNs, which require protein recoding for each new target site, CRISPR/Cas9 can be designed to target any genomic site by simply altering the protospacer sequence of gRNA [92]. Moreover, the CRISPR/Cas9 system can facilitate genome editing at multiple locations by encoding multiple RNA guide sequences into a single CRISPR array [24].

In addition, curative therapies, which are one-time treatments curtailing the symptoms of a disease permanently or semi-permanently, are gaining prominence in precision medicine to treat rare inherited disorders. One example is *Maino *et al*.'s* recent study that used the CRISPR/Cas9 system to correct Duchenne muscular dystrophy (DMD) tandem duplication mutation in mouse models [93]. The study used an sg-RNA and a Cas9 system to remove the DMD duplication mutation and restore full-length dystrophin expression in the mice [93].

Recently, the CRISPR system has also been used for editing mouse zygotes [94, 95]. The CRISPR/Cas9 components were shown to be delivered into the zygotes via microinjection or electroporation to produce genetically modified offspring [94]. Although the future use of the system in zygote editing seems promising, ethical concerns regarding the use of CRISPR in human germline editing remain. In this context, few recent studies have revealed unintended genome editing outcomes in CRISPR/Cas9–targeted human preimplantation embryos [96]. The study observed loss of heterozygosity and segmental loss and gain of chromosome 6 in the edited human embryo cells [96]. Yet another recent study evaluated the repair outcomes of a Cas9-induced double-stranded break on the paternal chromosome carrying a frameshift mutation that causes blindness. The most common repair mechanism was microhomology-mediated end joining, which successfully restores the reading frame in the embryos [97]. Although the Cas9 successfully repaired DSBs to some extent, more than half of the DSBs that lead to indels and chromosomal loss remained unrepaired [97].

The first-ever Chinese clinical trial of the CRISPR/Cas9 system involved injecting genetically modified cells into a patient with lung cancer. In this case, the CRISPR/Cas9 system was used to delete the *PD-1* gene that downregulates the immune system response [98]. After the first clinical trial, many clinical trials (in vivo and ex vivo) utilizing the CRISPR/Cas9 system to treat human pathologies emerged (reviewed in [86]).

Apart from gene editing, the CRISPR/Cas9 system has been used in antiviral applications. During the ongoing COVID-19 pandemic, rapid and simple point-of-care testing is urgently needed. COVID-19 fits in the field of precision medicine owing to the diverse nature of disease symptoms across individuals. The CRISPR/Cas complex can be used for nucleic acid detection of SARS-CoV-2 as the complex can bind to the target region and cleave the nearby reporter nucleic acid constructs, thus indicating the presence of a viral nucleic acid [99]. Moreover, many studies have used CRISPR-based diagnostic tools to detect SARS-CoV-2 via CRISPR-Cas effector Cas13, a protein that can target RNA and, therefore, can detect SARS-CoV-2, a single-stranded RNA virus [100, 101].

Although CRISPR/Cas9 is an efficient tool for high-throughput screening, large-scale genome-wide screening using the CRISPR system needs much improvement in specificity, off-target effects, and analytical methods. In addition, more efficient delivery systems must be developed to increase the CRISPR/Cas9-system screening efficiency of the immune system genes in vivo. Thus, the future of CRISPR/Cas9 in precision medicine looks promising so long as the ethical concerns arising from the adverse effects of the system are considered and adequately addressed.

## CRISPR/Cas9 and artificial intelligence

**Fig. 5:**
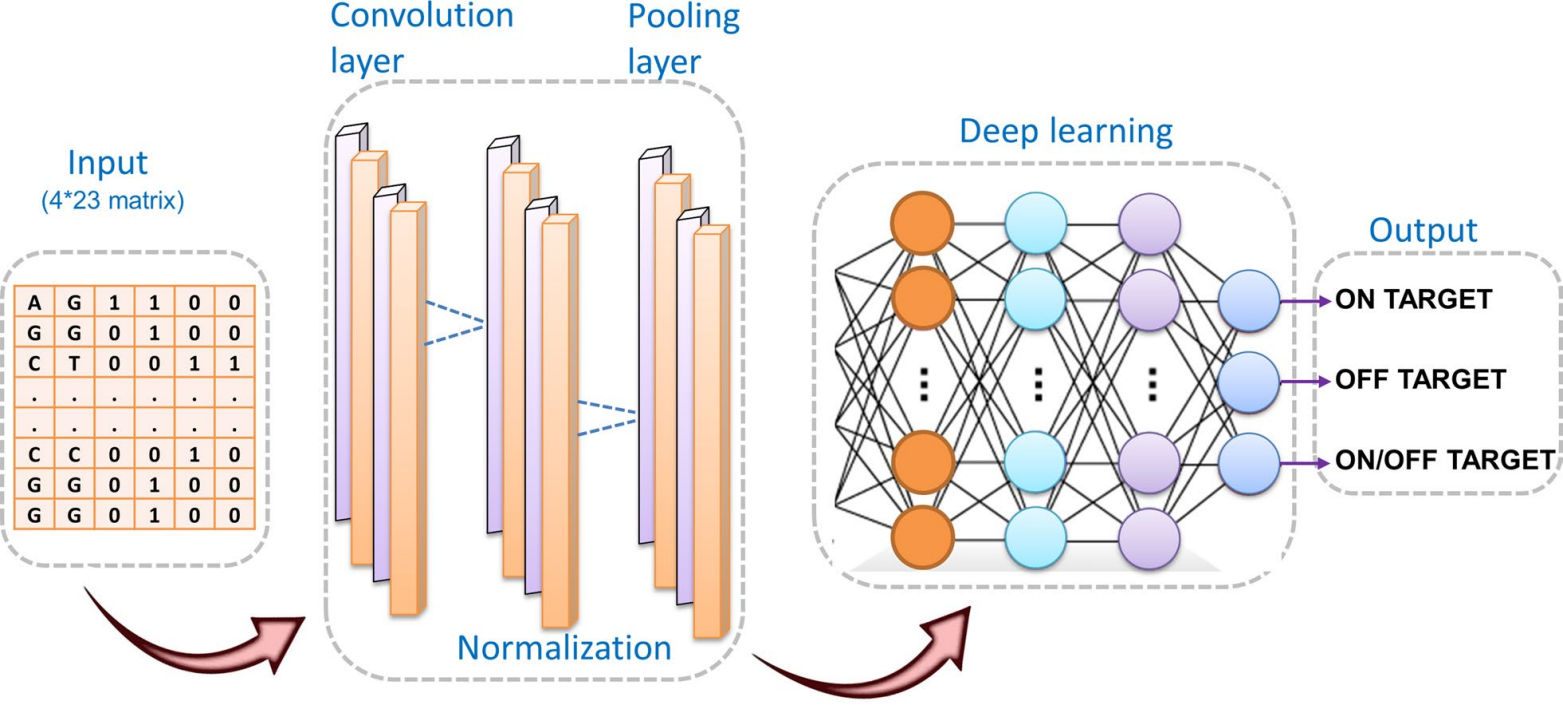
CRISPR/Cas9 deep learning architecture. Artificial intelligence-based deep learning model architecture showing different steps for predicting on/off-targets in the CRISPR/Cas9 system. The model takes a 4 × 23 code matrix corresponding to 4 nucleotides of 23 sequence length as input. The input is passed to the convolutional layer for obtaining sgRNA-DNA matching information by applying different filters of varied sizes. The information is passed for batch normalization to reduce the effect of internal covariates. A pooling layer is connected to the normalization layer which filters out the non-informative values. The result of pooling layer is converted into a single vector by flattening which is connected to the fully connected layer for final model classification

Numerous gRNA features or modulators have been identified that affect the cleavage efficiency of gRNA resulting in off-target effects. These include protospacer adjacent motifs (PAMs), gRNA sequence motifs, nucleotide usage in gRNA, position-specific nucleotide composition, secondary structure, and epigenetic features [102]. With the in silico gRNA design accounting for a crucial parameter in a successful gene edit by the CRISPR-Cas9 system, current efforts are now more focused on refining the gRNA design with improved on-target efficacy and minimum off-target effects [103]. As such, recent studies have described various algorithms that may help predict the various on-target and off-target effects of the CRISPR gRNAs, thus improving the respective activity and specificity of the CRISPR-Cas9 system [104]. These prediction tools have immensely assisted in improving the applications and success rates of the CRISPR system. However, the efficacy of gRNA is dependent on the interactions among various factors, including cellular environment, experimental conditions, gRNA, and target sequence, which can be overcome by machine learning (ML) based algorithms. The ML models are trained using existing datasets and can be used to predict the on/off-target effects of testing datasets. The present ML models are based on three methods: (a) regression-based methods [105–109], (b) classification-based methods [109–113], and (c) ensemble-based methods [114, 115]. The main differences in different categories of current ML tools are in the features they use and the presentation of the target site in model [102]. Advanced ML has also enabled the deployment of deep learning (DL) methods, such as artificial neural networks (ANNs) for the CRISPR-Cas9 system allowing high-precision target predictions. DL models in the CRISPR/Cas9 system comprise multiple layers of interconnected compute units. The algorithm takes the encoded gRNA-DNA sequence of length 23 in the matrix format as input. The convolutional layer applies various filers of different sizes to the input matrix. The next layer performs batch normalization to the resultant data from the previous layer to boost learning and avert over-fitting. The third pooling layer applies further filtration to the normalized data. The output of the pooling layer passes through different dense layers, and the neurons in these dense layers are fully interconnected. The last dense layer passes the result to the stop layer and predicts whether the input is off-target or on-target. The model architecture of a DL model is represented in (Fig. 5). Artificial Intelligence–based Machine and Deep Learning (MDL) methods are now gaining immense importance in gRNA design and CRISPR applications. The method uses algorithms based on the ever-increasing gene editing datasets reported globally and assists in predicting CRISPR gRNA activity and specificity scores [103]. Moreover, compared to experimental detection tools such as GUIDE-seq [116], HTGTS [117], or IDLV [118], the MDL-based methods are more efficient and cost-effective. A few examples of MDL-based algorithms that have been developed over the past few years to predict CRISPR on-target efficacy include CRISPRater [107], CRISPRScan [119], Azimuth 2.0 [120], TUSCAN [121], DeepCRISPR [122], DeepCas9 [123], WU-CRISPR [124], SgRNAScorer [113], CRISPRpred [125], DeepHF [126], CNN-SVR [127] and C-RNNCrispr [128].

It is pertinent to mention that the efficacy of MDL-based prediction tools is still not fully understood in different cell types and species [103]. Based on variability among various species, studies have now been focused on developing numerous species-specific software, such as fryCRISPR for Drosophila [129] and CRISPRscan for zebrafish [119]. Apart from predicting the on-target efficacy, algorithms have also been described that help predict the crucial off-target effects of the CRISPR-Cas9 system. These include the FlashFry [130], dsNickFury [131], CRISPR [132], DeepCRISPR [122], CNN_std [133], and Elevation [131] algorithms. The results of these and other studies have indicated that the off-target effects of CRISPR are not random and may be avoided by proper design of gRNA sequences [134]. Moreover, studies have shown that truncating the gRNA, especially the 5ʹ-end reduces the possible off-target effects [135].

The gRNA design algorithms, therefore, serve as a crucial milestone that may account for the proper application and efficient development of the CRISPR system shortly. Nevertheless, the existing models and algorithms suffer from drawbacks, including data imbalance and heterogeneity, lack of training datasets, and inefficiency across species, necessitating refinement before the gene editing system can be fully integrated into therapeutics. With the ever-evolving list of newer algorithms, the CRISPR/Cas9 system is expected to improve its increased on-target activity with corresponding minimum off-target effects, a crucial prerequisite in its clinical and therapeutic applications.

## Conclusion

The CRISPR/Cas9 system represents a revolutionary approach to gene editing and a potent tool in precision cancer medicine. The CRISPR/Cas9 cell screens and animal models have shown immense potential in discovering drug targets and cancer biomarkers, enabling more precise cancer treatments. The technology has enabled researchers to determine the frequency with which a tumor metastasizes, its origin, and its spread, making it possible to uncover changes in cancer that were otherwise undetected. CRISPR/Cas9 could also be a game-changer in the next generation of immuno-oncology cancer therapy. The precision and efficiency of multiplexed editing with CRISPR/Cas9 may overcome the primary challenges with the current generation of CAR-T therapies. The system has enabled the generation of off-the-shelf (allogeneic) CAR-T cells, providing significant gains over autologous (patient-derived) products. The system can be used to eliminate or insert genes to create new classes of CAR-T products with improved applicability to solid tumors. The integration of CRISPR-based assays with single-cell multi-omics approaches provides an array of applications that can be used to explore gene alterations and tumor heterogeneity. In addition, incorporating spatial transcriptomics with pooled CRISPR libraries can help understand the impact of genetic alterations on tumor microenvironment interactions [136]. Moreover, the application of CRISPR/Cas9 system in larger animal models will allow an efficient simulation of various human diseases, thus enriching the disease model resource bank.

Moreover, MDL-based algorithms have dramatically improved the system's efficacy concerning the reduced off-target effects, a crucial factor in broadening their application in clinical therapeutics. Using species-specific CRISPR algorithms has further enhanced the system's effectiveness across species. Newer CRISPR systems may continue to astound us once further work is carried out, especially in the context of the well-designed clinical trials needed before the technology can be used for cancer treatment. We expect that in the coming decade, advancements in the next-generation gene editing technologies will expand the versatility of the CRISPR/Cas9 system and enhance its applicability in diverse biological systems, thus becoming an indispensable tool to uncover the complexity of different human diseases.

## Data Availability

Not applicable.

## Abbreviations

CRISPR: Clustered regularly interspaced short palindromic repeats
Cas9: CRISPR-associated protein 9
ZFNs: Zinc finger nucleases
TALENs: Transcription activator-like effector nucleases
DSBs: Double-stranded breaks
NHEJ: Non-homologous end joining
HDR: Homology-directed repair
NGS: Next-generation sequencing
CSC: Cancer stem cell
CrRNA: CRISPR RNA
tracrRNA: Transactivating cRNA
sgRNA: Single guide RNA
CRISPRi: CRISPR interference
CRISPRa: CRISPR activation
SPCas9: *Streptococcus pyogenes* Cas9
MLL-r: Mixed lineage leukemia-rearranged
TNBC: Triple-negative breast cancer
HCC: Hepatocellular carcinoma
MSI: Microsatellite instabilities
BBDIs: BET bromodomain inhibitors
ATR: Ataxia telangiectasia and Rad3-related
EML4: Echinoderm microtubule-associated protein-like 4
PD-1: Programmed cell death protein 1
ALK: Anaplastic lymphoma kinase
IDH1: Isocitrate dehydrogenase 1
IDH2: Isocitrate dehydrogenase 2
AML: Acute myeloid leukemia
HGSC: High-grade serous tubo-ovarian cancer
3D: Three-dimensional
TCR: T-cell receptor
CAR: Chimeric antigen receptor
NK: Natural killer
hiPSCs: Human-induced pluripotent stem cells
DMD: Duchenne muscular dystrophy
